# RRNPP-type quorum sensing affects solvent formation and sporulation in *Clostridium acetobutylicum*

**DOI:** 10.1099/mic.0.000916

**Published:** 2020-04-28

**Authors:** Ann-Kathrin Kotte, Oliver Severn, Zak Bean, Katrin Schwarz, Nigel P. Minton, Klaus Winzer

**Affiliations:** ^1^​ BBSRC/EPSRC Synthetic Biology Research Centre (SBRC), School of Life Sciences, University Park, The University of Nottingham, Nottingham, UK; ^†^​ Present address: Independent Commodity Intelligence Service, Bishopsgate, London, UK; ^‡^​ Present address: Singer Instruments, Roadwater, Watchet, UK; ^§^​ Present address: CHAIN Biotechnology Ltd, MediCity, Nottingham, UK; ^#^​ Present address: Azotic Technologies Ltd, BioCity, Nottingham, UK

**Keywords:** butanol, *Clostridium acetobutylicum*, RRNPP-type regulator, solventogenesis, sporulation, quorum sensing

## Abstract

The strictly anaerobic bacterium *Clostridium acetobutylicum* is well known for its ability to convert sugars into organic acids and solvents, most notably the potential biofuel butanol. However, the regulation of its fermentation metabolism, in particular the shift from acid to solvent production, remains poorly understood. The aim of this study was to investigate whether cell–cell communication plays a role in controlling the timing of this shift or the extent of solvent formation. Analysis of the available *C. acetobutylicum* genome sequences revealed the presence of eight putative RRNPP-type quorum-sensing systems, here designated *qssA* to *qssH*, each consisting of an RRNPP-type regulator gene followed by a small open reading frame encoding a putative signalling peptide precursor. The identified regulator and signal peptide precursor genes were designated *qsrA* to *qsrH* and *qspA* to *qspH*, respectively. Triplicate regulator mutants were generated in strain ATCC 824 for each of the eight systems and screened for phenotypic changes. The *qsrB* mutants showed increased solvent formation during early solventogenesis and hence the QssB system was selected for further characterization. Overexpression of *qsrB* severely reduced solvent and endospore formation and this effect could be overcome by adding short synthetic peptides to the culture medium representing a specific region of the QspB signalling peptide precursor. In addition, overexpression of *qspB* increased the production of acetone and butanol and the initial (48 h) titre of heat-resistant endospores. Together, these findings establish a role for QssB quorum sensing in the regulation of early solventogenesis and sporulation in *C. acetobutylicum *.

## INTRODUCTION

The strictly anaerobic bacterium *Clostridium acetobutylicum* is well known for its ability to convert sugars and starches into organic acids and solvents [1, 2]. During the first half of the last century, the organism was used for the large-scale industrial production of acetone and butanol, but the classical acetone–butanol–ethanol (ABE) fermentation process is not currently economically viable [3]. Thus, considerable efforts have been devoted to improving the organism’s performance through metabolic engineering [4]. However, our understanding of the organism’s physiology and metabolism, in particular the mechanisms that govern timing and extent of solvent formation, is still limited [5, 6].

In a typical *C. acetobutylicum* batch culture, acid and solvent metabolism are associated with different growth phases. During the exponential phase, a characteristic butyric acid fermentation is carried out, leading to the accumulation of butyrate and acetate in the culture medium. This poses a problem for the cells as the pH of the medium decreases and undissociated acids diffuse back into the cells. To avoid collapse of the proton motive force, *C. acetobutylicum* shifts its metabolism to solvent formation. In batch culture, this shift usually occurs during the transition to stationary phase and is accompanied by the partial uptake of the previously produced acids, resulting in a pH increase. These acids, together with the remaining sugars, are then converted to butanol, acetone and ethanol [1, 2]. However, solvents at high concentrations, in particular butanol, are toxic to the cells, too. The metabolic switch to solvent formation therefore leads to the initiation of yet another survival strategy: the formation of heat-resistant endospores. After solvent formation has been initiated and after cells have entered stationary phase, an intracellular starch-like storage compound termed granulose is transitorily formed and accumulates in the cytoplasm [2].

The regulatory mechanisms that govern acid and solvent metabolism, as well as sporulation, are subject to intensive research. The global transcriptional regulator Spo0A is known to be required for high solvent production in solventogenic *Clostridium* sp. and is also essential for the initiation of sporulation [7–9]. Other regulators implied in the regulation of fermentation metabolism are the global regulator CodY, a small regulatory RNA, *solB*, and the catabolite control protein CcpA [10–13]. Responsible for the Spo0A phosphorylation state and thus its activity are three orphan histidine kinases, as well as an intracellular kinase-like protein, which, however, acts as a phosphatase [14]. Unfortunately, none of the signals or cues activating or inhibiting these proteins are currently known, although intracellular accumulation of butyrylphosphate has been proposed as a possible physiological signal and Spo0A phosphodonor [15]. So while the general conditions for solventogenesis are well established, and considerable progress has been made in unravelling at least some of the regulatory networks involved, we are still largely ignorant of the cues and signals that ultimately control the initiation and extent of solvent formation, and of the pathways through which they mediate their effects.

We recently proposed that quorum-sensing systems may be operational in *C. acetobutylicum* and may play a role in regulating solvent metabolism [16]. Quorum sensing is a mechanism of cell–cell communication that relies on small, diffusible signal molecules often referred to as autoinducers. These molecules are secreted during growth, accumulate in the extracellular environment, and allow bacteria to coordinate gene expression with cell population density. In the *Firmicutes *, quorum-sensing systems are usually based on secreted autoinducing peptides (AIPs), which can be linear or cyclic, and sometimes contain post-translational modifications [17–19]. Relatively little is known about the operation of such systems in clostridial species, but we have previously hypothesized [16] that quorum sensing might play a role in the regulation of solventogenesis based on the following considerations. First, for the solventogenic *Clostridium saccharoperbutylacetonicum* an as yet unidentified, self-generated signal present in the supernatant of wild-type cultures was capable of inducing solvent formation in a ‘low-solvent’ mutant [20]. Second, in *C. acetobutylicum* and related ‘high-solvent’ producers such as *Clostridium beijerinckii *, solventogenesis during batch culture growth is usually initiated at high cell densities. Third, genome sequencing has revealed a large number of putative quorum-sensing systems within the genus *Clostridium *, including all currently sequenced solvent-producing species ([21] and unpublished data from this laboratory). Furthermore, a novel polyketide signal, clostrienose, has recently been shown to affect granulose formation, sporulation and, to a smaller degree, butanol production [22].

We therefore investigated the role of a putative *agr*-type quorum-sensing system in *C. acetobutylicum* ATCC 824*,* which we showed to be functional and involved in the regulation of sporulation and the production of granulose [16]. However, the formation of organic acids and solvents from glucose was unaffected in mutants in which this system had been inactivated, suggesting that it played no role in the regulation of fermentation metabolism.

Interestingly, the *C. acetobutylicum* genome has been reported [23] to encode two proteins that bear resemblance to what is now known as the RRNPP family of quorum-sensing regulators [18, 19, 24]. This protein family derives its name from its best-studied members, i.e. Rap, Rgg, NprR, PlcR and PrgX, and is characterized by the presence of tetratricopeptide repeat (TPR) domains responsible for promoting protein–protein interaction. It comprises all currently known Gram-positive cytoplasmic quorum-sensing regulators that bind directly to their cognate signalling peptide. This signalling peptide is (generally) derived from the C-terminal part of a precursor peptide that is exported and further processed to its mature, active form [18, 19, 24]. The mature signalling peptide is transported back into the cells by oligopeptide permeases belonging to the family of ATP-binding cassette (ABC) transporters. Thus, the uptake of these signalling molecules is an ATP-consuming process [23]. Apart from the Rap proteins, all other currently identified RRNPP-type regulators, including the two proposed *C. acetobutylicum* homologues (CA_C186 and CA_C3694), possess helix–turn–helix (HTH) motifs and are either known or likely to be transcriptional regulators that become activated or inhibited upon binding their cognate signalling peptide [18, 19, 24].

Here, we report the discovery and mutational screening of eight putative RRNPP-type quorum-sensing systems in *C. acetobutylicum* ATCC 824 and the more detailed characterization of one of these systems, QssB.

## RESULTS

### Identification of eight putative RRNPP-type quorum-sensing systems in *C. acetobutylicum*

Using the two previously identified *C. acetobutylicum* homologues [23] and other experimentally confirmed HTH-containing RRNPP-type regulators such as PlcR and NprR in blastp searches, a total of 11 putative RRNPP-type regulators genes were identified in published *C. acetobutylicum* genomes (strains ATCC 824, DSM 1731 and EA 2018). The locus tags for these genes in the ATCC 824 strain were CA_C0186, CA_C0324, CA_C0957, CA_C0958, CA_C1043, CA_C1214, CA_C1949, CA_C2490, CA_C3694, CA_P0040 and CA_P0149. Most of them were annotated as either hypothetical proteins or regulators of the Xre family containing TPR domains, with CA_C0186 and CA_C3694 representing the previously identified homologues [23]. In the current version of the ATCC 824 genome (NC_003030.1), CA_C3694 is annotated as a pseudogene in which the HTH motif and TPR domain-encoding parts of an RRNPP-type regulator are separated by a stop codon. However, in the genomes of the DSM 1731 and EA 2018 strains, these domains are encoded by a single gene. We therefore compared the published ATCC 824 sequence to that obtained for our version of this strain [25] and also found the two domains to be encoded by a single gene. In the original ATCC 824 sequence, the insertion of a guanine at position 357 had shifted the reading frame so that a stop codon appeared to terminate CA_C3694 translation after 360 bp. The corrected sequence was identical to that in the EA 2018 and DSM 1731 strains [26, 27].

**Fig. 1.**
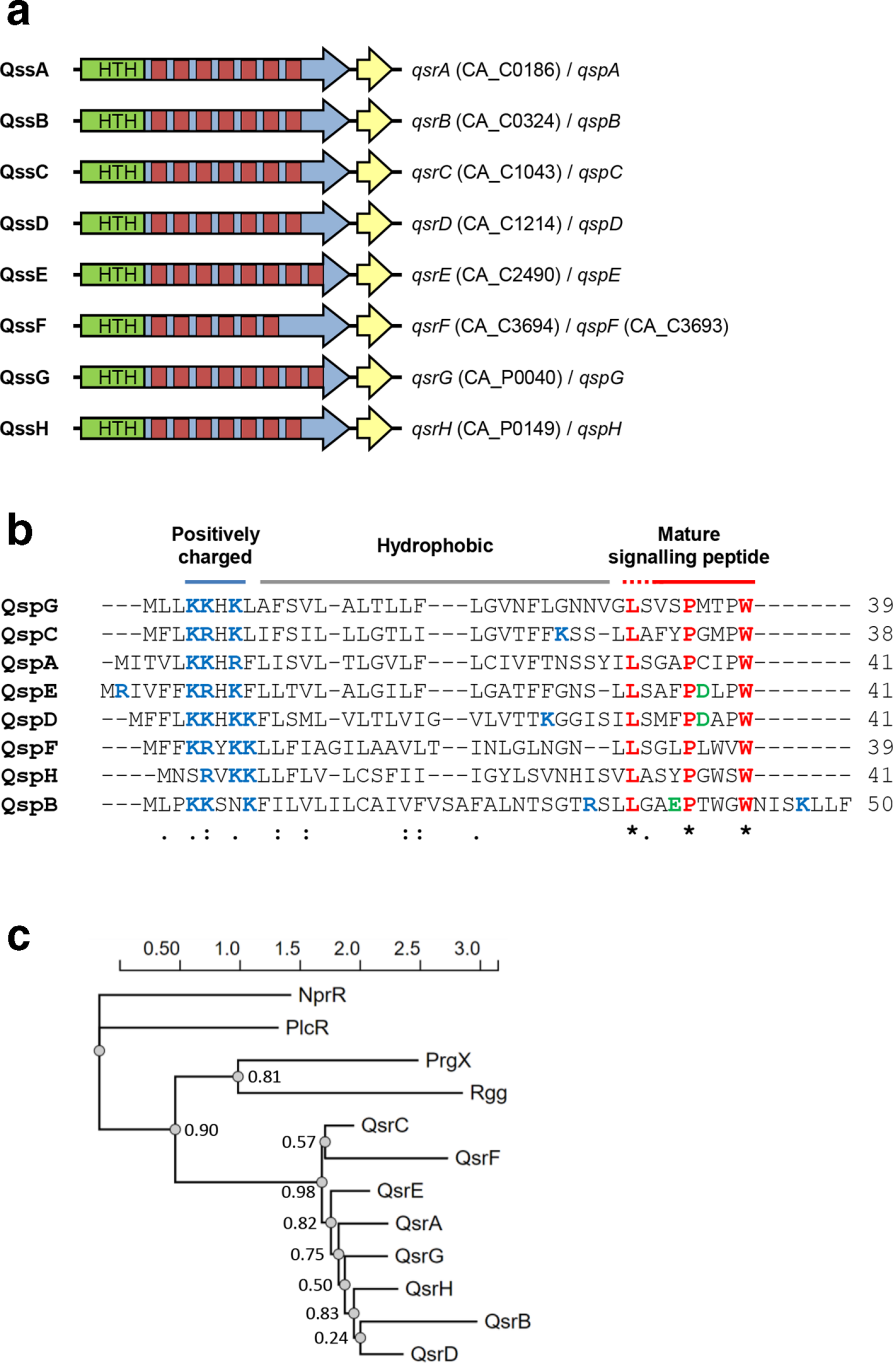
Schematic representation of *C. acetobutylicum* RRNPP quorum-sensing gene clusters (a), alignment of putative signalling peptide precursor sequences (b), and phylogeny of Qsr proteins (c). (a) Eight RRNPP quorum-sensing gene clusters have been identified (QssA to QssH), each encoding an RRNPP-type regulator (QsrA to QspH, large arrows) and a signalling peptide precursor (QspA to QspH, short yellow arrows). The locus tags for each system are provided, where available. Regions encoding a helix–turn–helix motif (HTH, green) and tetratricopeptide repeat domains (red) are indicated. (b) The clustal Omega amino acid sequence alignment shows the eight predicted Qsp proteins. Red font indicates amino acids that are 100 % conserved; blue and green fonts indicate positively (K, R) and negatively charged (d, e) amino acids, respectively. Identical (*), conserved (:) and semi-conserved substitutions (.) are shown. Numbers indicate the length of the different precursor proteins. Positively charged, hydrophobic and predicted signalling peptide-encoding regions are indicated with blue, grey and red lines, respectively. (c) Maximum-likelihood phylogeny of *C. acetobutylicum* Qsr proteins and representatives of the NprR (AFV16330.1), PlcR (AAV51966.1), PrgX (ARF06186.1) and Rgg (ABF31594.1) subgroups of RRNPP family proteins generated by PhyML. Numbers next to nodes indicate branch support values. The scale bar indicates the number of amino acid substitutions per site.

To establish a putative role in quorum sensing, the regions flanking the above regulator genes were analysed for the presence of short open reading frames (ORFs) encoding putative quorum-sensing peptide precursors. For established RRNPP-type systems, these precursors consist of a positively charged N-terminus, followed by a hydrophobic region (together forming a signal peptide sequence, required for peptide export) and a C-terminal part containing the actual autoinducing peptide [18, 23]. Short ORFs fulfilling the above criteria could be identified downstream of CA_C0186, CA_C0324, CA_C1043, CA_C1214, CA_C2490, CA_C3695/CA_C3694, CA_P0040 and CA_P0149 (Fig. 1a). Only one of these ORFs (CA_C3693) was annotated in the ATCC 824 genome sequence. The eight aforementioned regulator genes were therefore designated quorum-sensing regulators A to H (*qsrA* to *qsrH*), and their cognate quorum-sensing peptide (Qsp)-encoding genes *qspA* to *qspH*, respectively. Together they were referred to as quorum-sensing systems A to H (QssA to QssH).

Comparison of the identified Qsp sequences revealed that they were of similar length (39–50 amino acids) and displayed low to moderate sequence conservation (Fig. 1b) with identities and similarities of between 12–53 % and 33–64 %, respectively. Only three amino acid positions at the C-terminal end were conserved in all Qsp proteins, representing a leucine, a proline and a tryptophan (Fig. 1b). The latter formed the C-terminal amino acid in all putative Qsp proteins, with the exception of QspB, which was extended by an additional seven amino acids.

The deduced Qsr sequences were also of similar length and showed sequence identities and similarities of between 31–55 % and 46–63 %, respectively. The N-terminal regions, which contained the predicted Xre-type HTH motif, represented the most highly conserved parts of the Qsr proteins. Using TPRpred [28], the remaining parts of the Qsr proteins were predicted to each possess seven putative TPR domains, with the exception of QsrF (six domains) and QsrE and QsrG (eight domains). Comparison with the HTH-domain containing RRNPP-type regulators Rgg, NprR, PlcR and PgrX revealed low identity and similarity values (below 18 and 29 %, respectively), with very few universally conserved amino acid positions, again mainly positioned in the predicted HTH domains of the proteins (data not shown). Accordingly, maximum-likelihood phylogeny revealed that the eight Qsr proteins formed a cluster that was distinct from those formed by the other HTH-containing RRNPP family regulators (Fig. 1c).

Analysis of available genome sequences revealed the presence of similar systems in other members of the class *Clostridia,* notably the solventogenic *C. saccharoperbutylacetonicum* strain N1-4, the genome of which was found to contain five putative RRNPP-type gene clusters (Fig. S1, available in the online version of this article). However, no such systems (i.e. containing both regulator and peptide) could be identified in the closely related *C. beijerinckii *.

### Insertional inactivation of *qsr* genes using ClosTron technology

Using ClosTron technology [29], all eight identified *qsr* genes were insertionally inactivated in the ATCC 824 strain. Correct insertion of *ermB*-carrying introns into the target genes was confirmed by PCR screens and sequencing of the obtained PCR products as described previously [16].

The chosen ClosTron insertion sites were located within the putative HTH-encoding region of the *qsr* genes (see the Methods section), thus ensuring that no active DNA-binding proteins could be formed. For each gene, at least three independently derived ClosTron clones were isolated and further characterized.

### Phenotypic screening of *qsr* mutants to identify systems of interest

The obtained *qsr* mutants were phenotypically characterized with respect to growth, colony morphology, starch degradation, granulose formation, sporulation and solvent formation.

When cultured in supplemented clostridial basal medium (CBMS), several mutants showed minor differences in their growth kinetics when compared to the wild-type (Fig. S2). Under the conditions employed, wild-type cultures reached an OD_600_ of 2.6 after 9 h, followed by a transient OD_600_ decrease to 1.4 (24 h) before reaching the final maximum OD_600_ of 3.1 (48 h). Concurrent with the transient decrease in OD_600_, the cultures began to appear more viscous, potentially due to accumulation of exopolysaccharide. Similar profiles were observed for the mutant strains, although *qsrF* and *qsrG* mutants reached lower final ODs after 48 h (1.92 and 1.03, respectively), *qsrC* and *qsrD* mutants grew more slowly, and *qsrB* mutant cultures already showed the transitory OD_600_ decrease and viscosity increase after 12 h. The low final OD_600_ of the *qsrG* mutant presumably reflected the strain’s tendency to form large cell aggregates.

The ability to degrade starch was not affected in any of the mutants and granulose formation appeared to be similar to that of the wild-type (data not shown). Interestingly, however, after 24 h of growth on solid *Clostridium* growth medium (CGM) plates the *qsrB* mutants were observed as forming larger colonies (2.0 mm) when compared to the wild-type (1.3 mm), a difference that was statistically significant (*P*<0.00001; Table 1).

**Table 1.** Colony size of *C. acetobutylicum* parent strain and *qsrB* mutants on CGM after 24 h

Strain	Mean colony size in mm ±sd (*n*=20)
* C. acetobutylicum* ATCC 824	1.3±0.49
*C. acetobutylicum qsrB*::CT*ermB*	2.0±0.24*****
* C. acetobutylicum* ATCC 824 pMTL 85141	1.2±0.32
*C. acetobutylicum qsrB*::CT*ermB* pMTL85141	1.5±0.35**
*C. acetobutylicum qsrB*::CT*ermB* pMTL85141-*qsrB*	1.0±0.28†

*****Significantly different from the vector-free wild-type control at *P*<0.00001; *n*=20.

**Significantly different from the vector-carrying wild-type at *P*<0.01.

†Not significantly different from the vector-carrying wild-type.

Microscopic examination of CBMS-grown cultures revealed no noticeable changes in the number of endospores formed by *qsr* mutants when compared to the wild-type. However, a significant threefold reduction (*P*=0.036) was observed for *qsrG* mutants when a more quantitative procedure was used, i.e. when the number of heat-resistant spores in a given culture volume was determined after 7 days of culture [Table S1; more precisely, this procedure quantifies the number of heat-resistant colony-forming units (c.f.u.) as a measure for spores that can germinate and grow after heat treatment at 80 °C for 10 min].

The ability of *qsr* mutants to form butanol, acetone and ethanol was also assessed during early (24 h) and late (120 h) solventogenesis. According to their butanol production profiles (Fig. S3), *qsr* mutants could be grouped into four categories: (i) early and late butanol titres similar to the wild-type: *qsrC* and *qsrD* mutants; (ii) increased butanol titres during early solventogenesis: *qsrB* mutants; (iii) decreased butanol titres during early solventogenesis: *qsrA* and *qsrE* mutants; (iv) decreased butanol titres during late solventogenesis: *qsrF*, *qsrG*, *qsrH*. Generally, changes in butanol titres were mirrored by the corresponding acetone concentrations. However, this was not always the case for ethanol. For instance, final (120 h) ethanol titres were significantly increased for the *qsrB* and *qsrE* mutants and early (24 h) titres in *qsrA* and *qsrE* mutants were comparable to those of the wild-type (Fig. S3).

### QsrB represses solvent formation

**Fig. 2.**
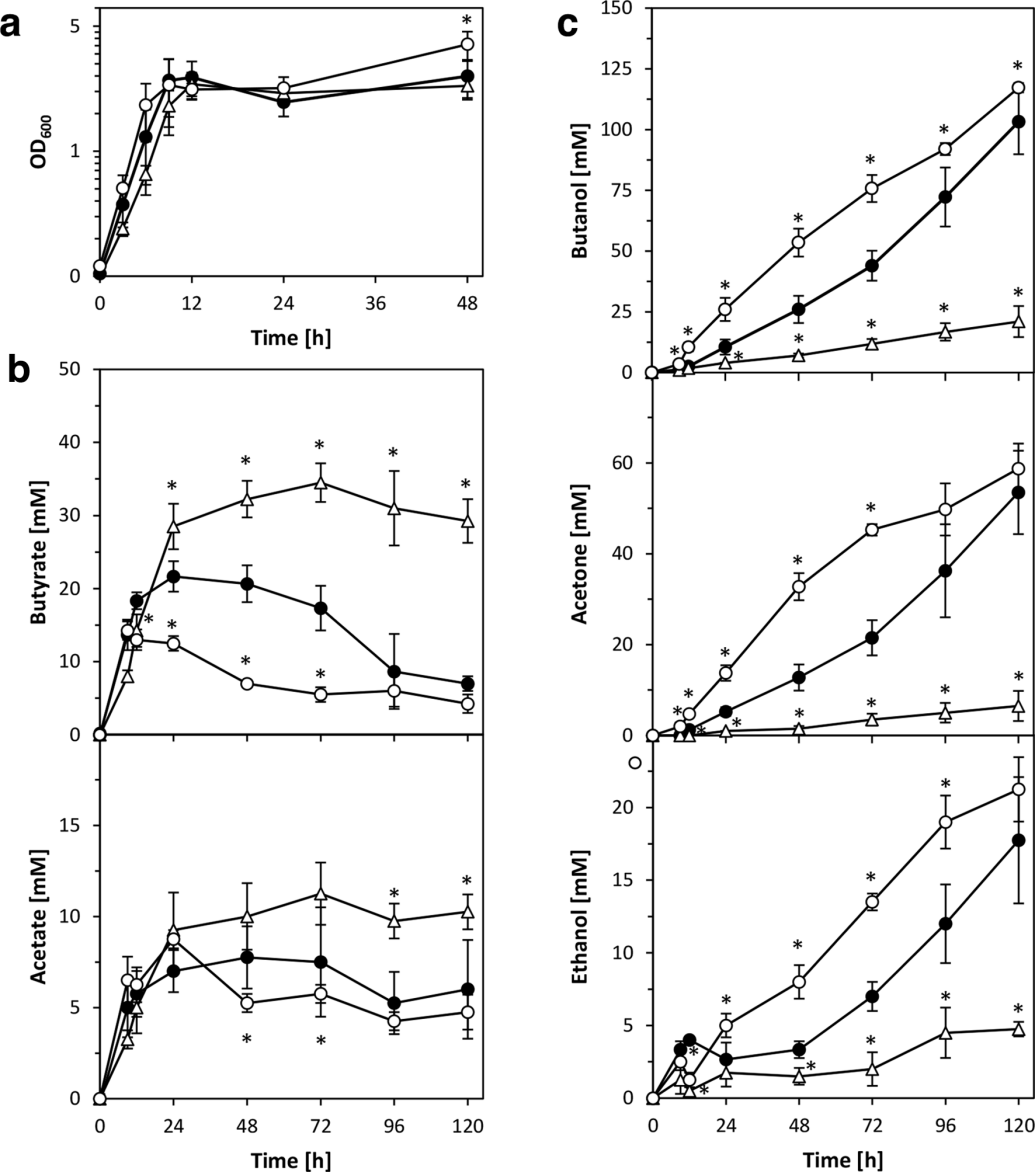
Fermentation profile of *C. acetobutylicum* wild-type, *qsrB* mutants and genetically complemented *qsrB* mutants. Growth (a) and production of acids (b) and solvents (c) were compared for the ATCC 824 parent strain containing the empty pMTL85141 vector (closed circles), the *qsrB* mutants containing the empty pMTL85141 vector (open circles) and the *qsrB* mutants containing the complementation plasmid pMTL85141-*qsrB* (open triangles). Data represent the mean of four independent CBMS cultures with error bars indicating the standard deviation. Significant differences (*P*≤0.05) compared to the wild-type are indicated by an asterisk next to the relevant data point.

Following the initial phenotypic screening, the *qsrB* mutants were selected for a more thorough characterization as they exhibited a number of relevant phenotypic changes, including growth profile, colony size/morphology and solvent production. Particularly relevant from a biotechnological perspective was the increased production of butanol during early solventogenesis. More detailed fermentation profiles were therefore generated, with samples taken at regular intervals during a 120 h growth experiment. These profiles confirmed the increased production of solvents during early solventogenesis and also revealed that, after entry into stationary phase, *qsrB* cultures contained lower concentrations of butyric and acetic acid (Fig. S4). To obtain ultimate proof that *qsrB* inactivation was responsible for the observed phenotypes, the obtained *qsrB* mutants were genetically complemented. *qsrB* under control of its native promoter was cloned into the modular shuttle vector pMTL85141 [30] and the resulting pMTL85141-*qsrB* vector was used to transform the *qsrB* mutant strains via electroporation. As a control, unmodified pMTL85141 was also introduced into both wild-type and *qsrB* mutant strains. Indeed, complementation with plasmid-based *qsrB,* but not the empty shuttle vector, reversed the effects of *qsrB* inactivation, i.e. it reduced the production of all three solvents, increased the production of acetic and butyric acid, and reduced colony size (Fig. 2, Table 1). However, while colony size was restored to approximately wild-type levels, the metabolic changes resulting from the complementation were more drastic, i.e. solvent production by the complemented *qsrB* mutants was significantly lower, and acid production markedly higher, than observed for the wild-type. It was hypothesized that this was caused by the presence of multiple *qsrB* copies in the complemented mutants, leading to *qsrB* overexpression. Very similar results were obtained when the experiments were repeated using the overexpression vector pMTL85143, which carries a strong constitutive ferredoxin gene promoter to drive the expression of the inserted *qsrB* gene (data not shown). Expression of *qsrB* in the ATCC 824 wild-type using the pMTL85141-*qsrB* and pMTL85143-*qsrB* plasmids yielded similar fermentation profiles to those observed for the complemented *qsrB* mutant, with increased production of acids and considerably reduced solvent formation (Table 2).

**Table 2.** Effect of *qsrB* overexpression on the fermentation product profile of wild-type *C. acetobutylicum* ATCC 824 after 120 h growth in CBMS

Product	* C. acetobutylicum *			
(mM)	pMTL85141	pMTL85141-*qsrB*	pMTL85143	pMTL85143-*qsrB*
Butyrate	7±1	36±3****	13±13	48±15****
Acetate	6±3	14±1**	25±18	35±16*
Butanol	103±15	15±3****	86±23	9±4***
Acetone	54±4	4±1****	38±18	1±1**
Ethanol	18±4	4±3**	15±9	1±1*

*, **, *** and **** indicate significant differences from the vector-carrying wild-type at *P*<0.05, *P*<0.01, *P*<0.001 and *P*<0.0001, respectively; *n*=3.

### Overexpression of *qsrB* reduces spore formation

**Fig. 3.**
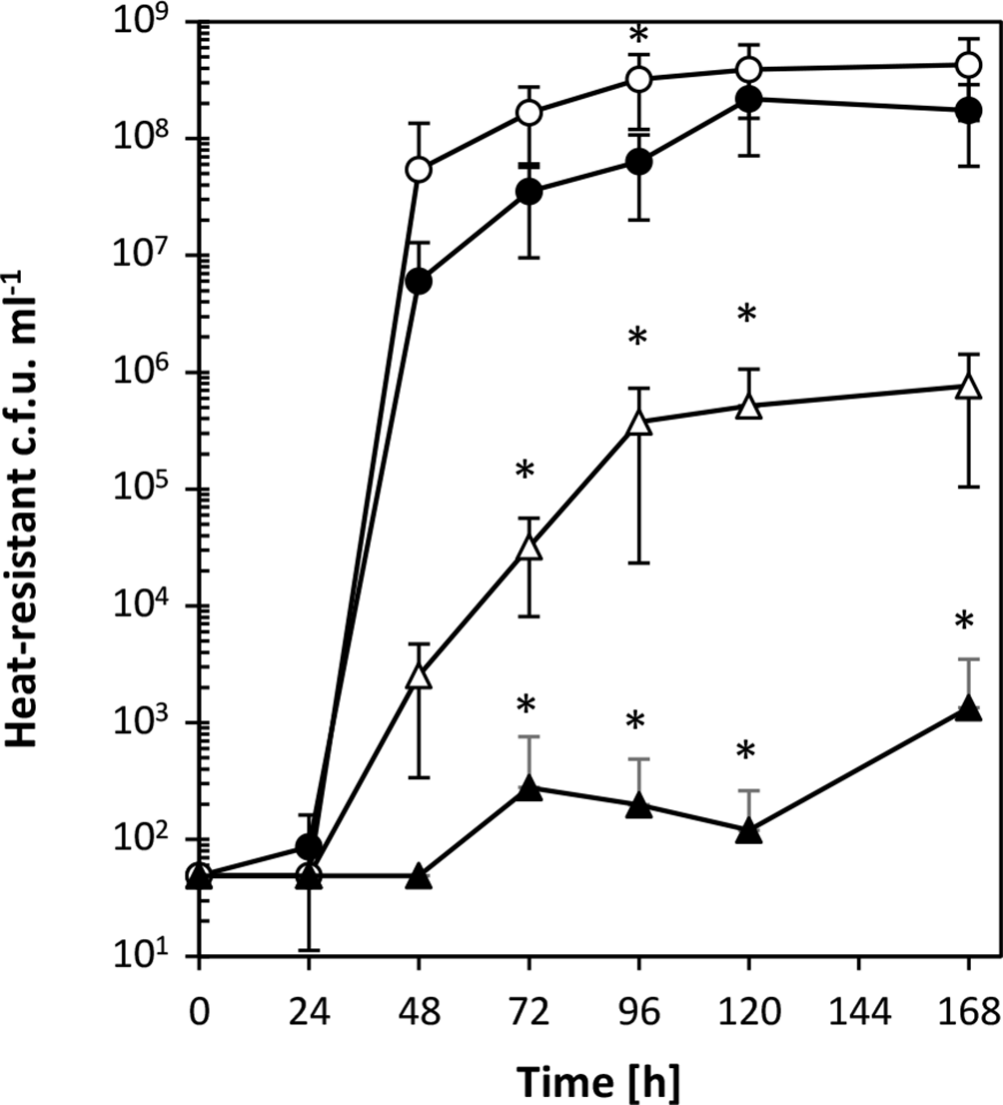
Effect of *qsrB* deletion and overexpression on sporulation. The ability to sporulate in CBMS was assessed for the ATCC 824 parent strain containing the empty pMTL85141 vector (closed circles), the *qsrB* mutants containing the empty pMTL85141 vector (open circles), the *qsrB* mutants containing the pMTL85141-*qsrB* complementation plasmid (open triangles) and the ATCC 824 parent strain containing the pMTL85141-*qsrB* complementation plasmid (closed triangles). Sporulation efficiencies were assessed by determining the number of heat-resistant endospores produced at the indicated time points. Data represent the mean of four independent CBMS cultures with error bars indicating the standard deviation. Only the upper half of the error bar is shown in cases where the lower half extends beyond 10^1^. Significant differences (*P*≤0.05) compared to the vector-carrying wild-type and vector-carrying *qsrB* mutant are indicated by an asterisk next to the relevant data point.

Given the drastic effects that *qsrB*-carrying plasmids had on acid and solvent formation, the number of heat-resistant endospores formed by the complemented *qsrB* mutants and *qsrB*-overexpressing wild-type were also assessed. These experiments revealed that in the presence of pMTL85141-*qsrB* both strains showed strongly reduced spore production (Fig. 3). Furthermore, while after 120 and 168 h there was no statistically significant difference in spore counts between wild-type and *qsrB* mutants both carrying the empty pMTL85141 control plasmid, the latter reached final spore levels earlier than the wild-type. This suggested earlier or more rapid sporulation in the absence of *qsrB*.

### Generation and characterization of *qspB* mutants

**Fig. 4.**
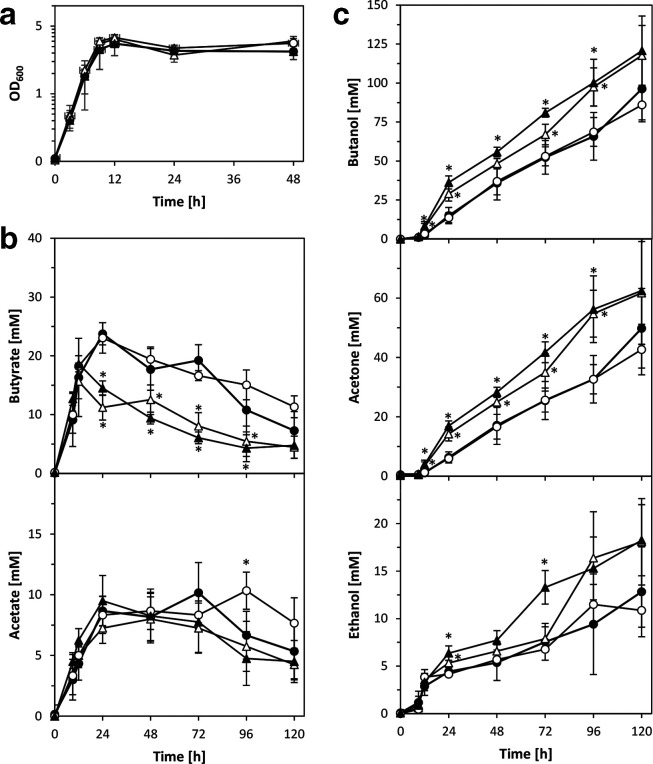
Fermentation profiles of *qspB*-overexpressing *C. acetobutylicum* wild-type and *qspB* mutants. Growth (a) and production of acids (b) and solvents (c) were compared for the ATCC 824 parent strain (closed symbols) and *qspB* mutant (open symbols) containing either the empty pMTL85143 vector (circles) or the overexpression plasmid pMTL85143-*qspB* (triangles). Data represent the mean of four independent CBMS cultures with error bars indicating the standard deviation. Significant differences (*P*≤0.05) compared to the vector controls are indicated by an asterisk next to the relevant data point.

**Fig. 5.**
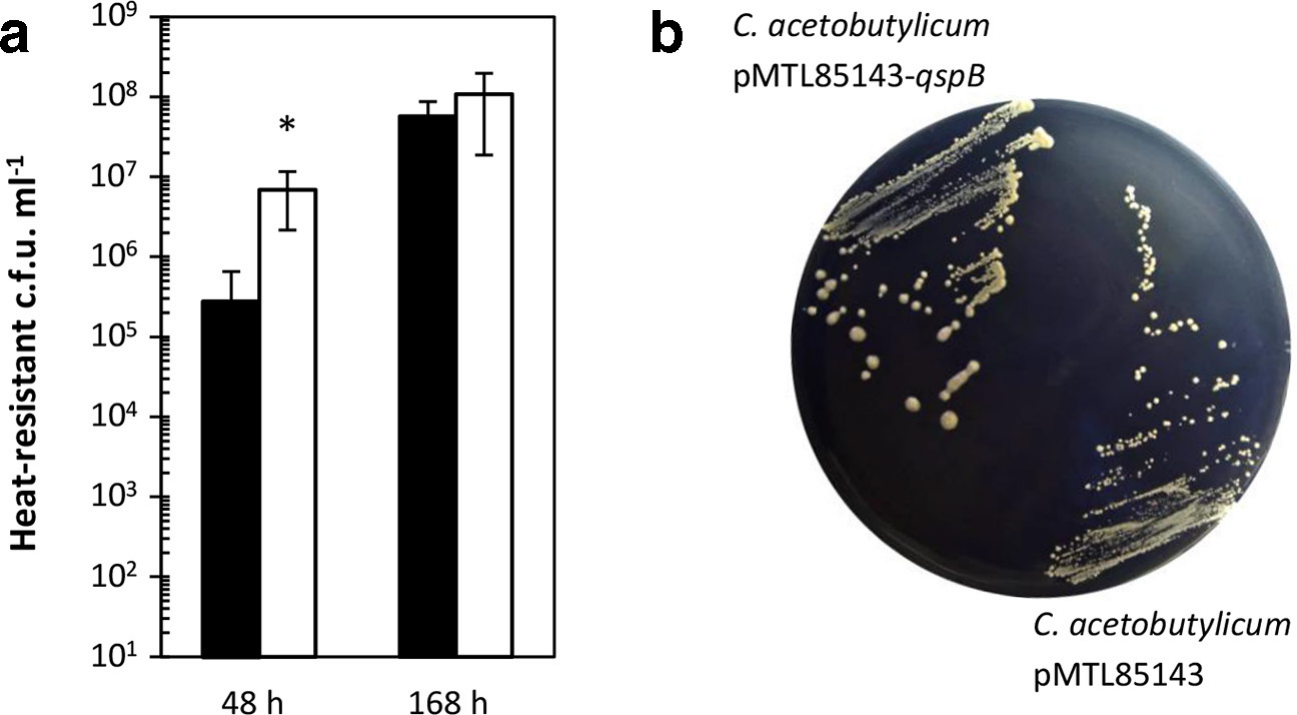
Effect of *qspB* overexpression on sporulation and colony size. (a) The ability to sporulate in CBMS was assessed for the *C. acetobutylicum* ATCC 824 parent strain containing the empty pMTL85143 vector (black bars) and the overexpression plasmid pMTL85143-*qspB* (white bars). Data represent the mean of four independent cultures with error bars indicating the standard deviation. Significant differences (*P*≤0.05) compared to the vector controls are indicated by an asterisk next to the relevant measurement. (b) 5-day-old colonies of *C. acetobutylicum* carrying the empty pMTL85143 vector (left) and overexpression plasmid pMTL85143-*qspB*, respectively, on CGM agar.

Based on the above results it appeared likely that *qsrB*-based quorum sensing contributes to the regulation of solvent formation and sporulation in *C. acetobutylicum *. To test this hypothesis, the role of the putative signalling peptide-encoding *qspB* gene, located downstream of *qsrB*, was investigated. Three independent *qspB* ClosTron mutants were generated and confirmed as described above for the *qsrB* mutants. While colony size, granulose formation and final spore levels were comparable to those of the wild-type, *qspB* mutant cultures showed reduced levels of acetone and butanol during late stationary phase, i.e. after 72 to 96 h. However, final (120 h) levels were comparable to those of the wild-type (data not shown). Introduction of the aforementioned shuttle vectors (pMTL85141 and pMTL85143; without insert) into *qspB* mutants and wild-type abolished the observed differences and led to indistinguishable solvent profiles (Fig. 4). Thus, conclusive genetic complementation experiments could not be conducted. However, when the *qspB* overexpression plasmid pMTL85143-*qspB* was introduced into both wild-type and *qspB-*deficient strains, solvent production increased significantly and butyrate concentrations during stationary phase were lower than in the control strains carrying the empty pMTL85143 plasmid. Acetate production, however, remained largely unchanged (Fig. 4b, c). Overexpression of *qspB* also increased colony size and led to an earlier increase in heat-resistant colonies, although the final spore levels appeared to be similar to those of the wild-type vector control (Fig. 5).

### *qspB*-encoded peptide fragments counteract QsrB-mediated repression of solventogenesis and sporulation

The similar phenotypes observed for *qsrB*-knockout and *qspB*-overexpressing strains suggested that either QspB or a QspB-derived quorum-sensing peptide might act to inhibit QsrB activity. To test the latter hypothesis, 13 peptide variants were synthesized, varying in length between 6 and 20 amino acids and covering various parts of the C-terminal region of QspB. These were then tested for their ability to restore butanol production in the *qsrB*-overexpressing strain *C. acetobutylicum* pMTL85141-*qsrB*. Cultures of this strain were supplemented with individual synthetic peptides at a final concentration of 10 µM and assayed for butanol formation after 120 h. Several of the exogenously added peptides were capable of restoring high-level butanol formation, whereas others had no discernible effect (Fig. S5). The latter group comprised all peptides terminating at amino acid 38 of the QspB sequence or starting at position 39, suggesting that the region conferring activity included amino acids upstream and downstream of these positions.

**Fig. 6.**
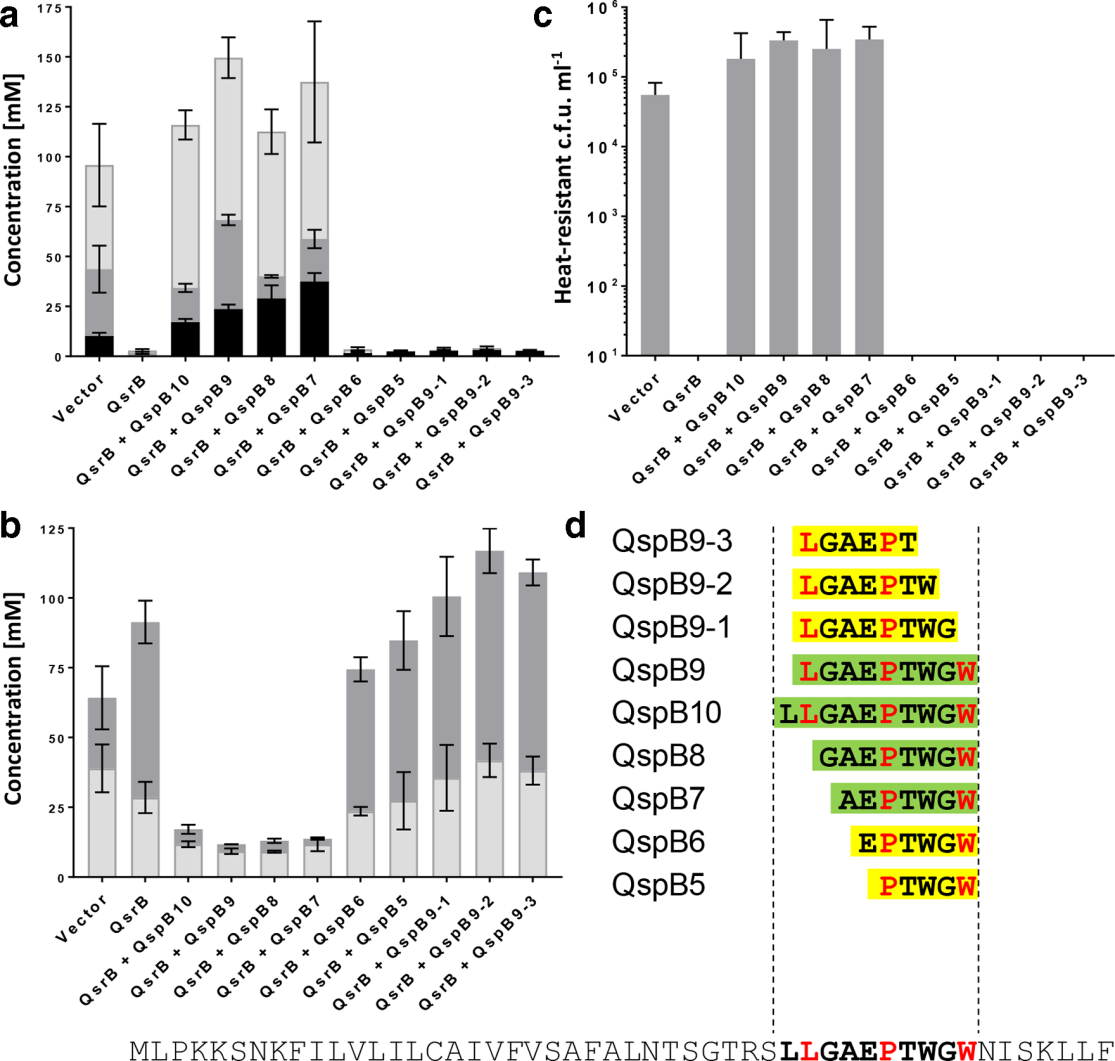
Synthetic peptides alleviate *qsrB*-mediated repression of solvent formation and sporulation. (a) Solvent titres: butanol (light grey), acetone (dark grey), ethanol (black). (b) Acid titres: butyrate (dark grey), acetate (light grey). (c) Spore titres (heat-resistant c.f.u.). (d) Synthetic peptides were dissolved in DMSO and individually added to CBMS cultures of *C. acetobutylicum* pMTL85143-*qsrB* after 4 h of growth to a final concentration of 10 µM. DMSO-only controls were performed for *C. acetobutylicum* pMTL85143 and *C. acetobutylicum* pMTL85143-*qsrB,* respectively. Cultures were grown for 5 days prior to analysis. *C. acetobutylicum* pMTL85143 vector control (Vector); *C. acetobutylicum* pMTL85143-*qsrB* cultures (QsrB). The presence of specific synthetic peptides as shown in (d) is indicated (+QspB). The complete QspB sequence is given at the bottom with the three conserved amino acid positions in the C-terminal region (leucine, proline, tryptophan) indicated by bold red lettering. Data represent the mean of three independent cultures with error bars indicating the standard deviation.

Based on these findings, additional peptide variants were designed, synthesized to a purity of >95 % and similarly tested. Interestingly, exogenous addition of QspB7, a peptide comprising only seven amino acids (AEPTWGW) and matching positions 37–43 of the QspB precursor, was capable of fully restoring butanol production in the *qsrB*-overexpressing *C. acetobutylicum* pMTL85143-*qsrB* strain (Fig. 6). The QspB7 sequence contained two of the three conserved amino acids present at the C-terminal end of all *C. acetobutylicum* Qsp proteins, i.e. proline and tryptophan (Fig. 1b). QspB-derived peptides capable of restoring high-level butanol formation were also found to dramatically increase acetone and decrease acid production when added to the *qsrB*-overexpressing strain (Fig. 6a, b). Furthermore, these peptides also restored high levels of sporulation (Fig. 6). The lowest QspB7 concentration that resulted in a statistically significant response (*P*<0.5) was 0.25 µM. At this concentration, butanol production by *C. acetobutylicum* pMTL85143-*qsrB* increased twofold.

## DISCUSSION

For many years, the precise molecular signals and mechanisms that trigger and control solvent formation in clostridia have remained elusive. Here we show that RRNPP-type quorum sensing is one of the contributing factors in *C. acetobutylicum* and suggest that this may also apply to other solventogenic species.

Bioinformatic analysis revealed the presence of at least eight RRNPP-type quorum-sensing systems in several sequenced strains of this bacterium, including two that had previously been proposed [23]. All eight were subjected to ClosTron mutagenesis, resulting in disruption of the postulated DNA-binding HTH motifs encoded in the 5′ regions of the respective *qsr* genes.

As a detailed analysis of all eight systems was beyond the scope of this study, a general phenotypic screen was carried out to identify mutants of interest, i.e. those showing clear phenotypic differences, in particular with relation to solvent metabolism. Intriguingly, inactivation of seven of these systems resulted in changes to the observed solvent profiles, although further studies will be necessary to establish whether these systems are directly involved in the regulation of solvent genes, or whether these are indirect effects resulting from other changes in growth, physiology and metabolic activity. Based on this initial screen, QssB was selected for a more detailed characterization, as it was the only system whose inactivation increased solvent formation and also affected multiple other phenotypes.

Taken together, our mutational analyses suggest that QssB plays a regulatory role during early solvent formation, controlling its extent and, potentially, precise timing. The data are consistent with QsrB acting as a repressor that is inactivated upon binding to its cognate QspB-derived signalling peptide. Alternatively, QsrB might exert its effects by positively regulating an unknown repressor of solvent formation. Both scenarios are supported by the findings that (i) solvent formation was increased by *qsrB* inactivation and strongly decreased by *qsrB* overexpression; (ii) overexpression of *qsrB* and *qspB* had opposing effects on solvent formation, sporulation and colony size; and (iii) repression of solvent formation and sporulation in *qsrB*-overexpressing cells could be overcome by adding short synthetic QspB-derived peptides to the culture medium. By contrast, the strong reduction of endospore formation seen for the *qsrB*-overexpressing strains might be an indirect consequence of the drastically reduced solvent metabolism. However, the Rap and NprR proteins of *Bacillus subtilis* and *Bacillus thuringiensis *, respectively, are known to have a role in the regulation of sporulation. Several Rap paralogues act as phosphatases that, in the absence of their cognate Phr signalling peptides, will dephosphorylate Spo0F and thus prevent Spo0A activation [18, 19, 24]. Similarly, in the absence of NprX signalling peptide, dimers of NprR will form and bind to Spo0F, thus physically preventing its phosphorylation. However, upon binding NprX, the NprR protein will adopt a tetrameric structure and transcriptionally activate genes required for necrotrophic growth [31]. Thus, the presence of a DNA-binding motif does not exclude a role in controlling Spo0A phosphorylation. It will be interesting to see how QsrB mediates its regulatory effects.

It is unclear why inactivation of the signalling peptide-encoding *qspB* gene had only limited effects during early solventogenesis, as under these conditions QsrB might be expected to repress, directly or indirectly, solvent formation in the absence of its cognate signalling peptide. An intriguing possibility could be that QsrB responds to more than one signalling peptide, i.e. lack of QspB may be compensated for by signalling peptides produced by the other quorum-sensing systems. This may also explain why the sporulation profile of the *qspB* mutant was so similar to that of the wild-type. The fact that *qspB* overexpression resulted in a small increase in sporulation may indicate that, under the employed culture conditions, wild-type signal molecule concentrations were not sufficiently high to completely inactivate the QsrB regulator at the time when this process was induced, consistent with the slight increase in sporulation observed for *qsrB* mutants.

Another interesting question is why quorum sensing control of solvent formation has evolved in *C. acetobutylicum *. A possible explanation could be that coordinated, population-wide responses are required to efficiently control the rapid production of toxic acids and, perhaps, at a later stage, solvents. For an optimal response, individual cells within the population may need to sense the density of acid-producing cells and this could be achieved by the extracellular accumulation of peptide signals such as those derived from QspB. Thus, before critical concentrations are irreversibly reached that lead to a fatal ‘acid crash’ [32], a population-wide decision is made to stop production and trigger a metabolic shift resulting in acid reuptake and solvent formation. Integrated with other relevant environmental information, this may enable the organism to maximize the number of cells that can enter solventogenesis and thus, eventually, sporulation to secure long-term survival. According to this hypothesis, uptake of acids and their conversion into solvents would be a social, cooperative trait, which is co-ordinately induced through quorum sensing at the appropriate population density.

It is evident from the above that sensing their density may help populations to shift from acid to solvent metabolism at an optimal stage of growth. It is less clear, however, why the organism contains such a large number of putative signalling systems, totalling 10 together with the previously described *agr* [16] and clostrienose systems [22]. Multiple systems are also present in many ubiquitous and/or metabolically versatile bacteria such as *B. subtilis* and members of the *B. cereus* group [18, 23, 33, 34].

It is therefore intriguing to see that two other, physiologically very similar, solvent-producing *Clostridium* species also contain a large number of putative signalling systems. Our analysis of the *C. saccharoperbutylacetonicum* genome revealed the presence of five complete RRNPP-type systems (Fig. S1) in addition to four potential *agr* systems (not shown). By contrast, *C. beijerinckii* NCIMB 8052 does not appear to contain complete RRNPP-type systems, but has six putative *agr* systems. Thus, while all three species are members of the genus *Clostridium sensu stricto*, carry out very similar ABE fermentations and, in the case of *C. beijerinckii* and *C. saccharoperbutylacetonicum *, are very closely related based on 16S rRNA sequence similarity, they differ considerably in terms of the cell–cell signalling systems they employ [35].

A possible explanation for employing multiple signalling systems may lie in the complex life cycle of these organisms, which not only involves a shift in fermentation metabolism, but also endospore formation and, under certain conditions, fruiting body formation [36]. These are all phenotypes for which quorum sensing control has been demonstrated in other species. A plausible mechanism for the emergence and accumulation of multiple quorum-sensing systems within a given lineage has recently been proposed by Even-Tov *et al*., who showed that this phenomenon may be driven by facultative cheating [33, 37]. This mechanism, which relies on a specific regulatory network structure, allows strains carrying an additional quorum-sensing system to exploit their ancestors when present at low frequencies and this may have contributed to the Rap–Phr expansion that has occurred independently in several species of the genus *Bacillus* [33, 34, 37]. Alternatively, it has been proposed that use of multiple signals may permit ‘combinatorial communication’, enabling bacteria to adjust gene expression to both social and physicochemical properties of their environment, particularly when accumulation of the individual signal molecules differs due to differences in half-life or diffusion constants [38]. Furthermore, non-combinatorial use of multiple peptide signals may simply enable cells to trigger responses at different density thresholds. To add to this complexity, the genomes of *C. acetobutylicum* and indeed all other members of the genus *Clostridium sensu stricto,* encode several orphan RRNPP-type regulators that are not flanked by small, signalling peptide-encoding genes. Whether genes of this type form part of a quorum-sensing systems or act independently of signalling peptides remains to be seen, but there is evidence to suggest that they, too, play major regulatory roles in their respective hosts. For instance, the CA_C0957/CA_C0958 regulators identified in this study were found to be important for solventogenesis and sporulation [39] and in *Clostridium difficile* another orphan RRNPP-type regulator was recently found to repress toxin production and motility, and upregulate sporulation [40].

The precise chemical nature of the Qsp-derived peptide signals produced by *C. acetobutylicum* remains to be established. In *B. subtilis *, some of the Phr peptides are derived from the C-termini of their respective precursor peptides, whereas others stem from internal regions [23]. A similar situation appears to be present in *C. acetobutylicum *. Our structure-activity analysis of QspB-derived peptide sequences clearly showed that biological activity is associated with a short internal sequence. However, for the other seven putative Qsp homologues, sequences corresponding to this region form the C-terminal end of the precursor peptide (Fig. 1).

Whereas the Phr signals produced by *B. subtilis* are pentapeptides, the *C. acetobutylicum* QspB peptide appears to be slightly larger, given that a heptapeptide was the shortest sequence for which biological activity was observed (Fig. 6). This heptapeptide, AEPTWGW, contained two of the three conserved amino acids present in the C-terminal region of all Qsp proteins, i.e. proline and tryptophan, whereas a slightly larger nonamer, LGAEPTWGW, showed similar activity but also contained the third conserved amino acid, leucine. This is similar to findings made for PlcR and its cognate heptapeptide signal, PapR, in the *B. cereus* group. Originally believed to be a pentapeptide due to its biological activity, the native PapR signal was later found to be a heptamer [41]. PapR peptides from different strains of this group show some variation in the first three N-terminal residues, whereas the C-terminal parts are relatively conserved [42]. Although the predicted *C. acetobutylicum* Qsp peptides show a larger degree of variation, the aforementioned proline (position −5) and tryptophan (position −1) are always present. Interestingly, the peptides produced by *B. subtilis* and the *B. cereus* group all contain charged amino acids [23, 42], whereas this is not the case for the majority of the predicted Qsp peptides. Only QspB, QspD and QspE are predicted to carry a negative charge, whereas all other Qsp peptides are highly hydrophobic. Whether and how this relates to their biological roles remains to be investigated.

In summary, we have identified multiple signalling systems in *C. acetobutylicum *, at least one of which plays a role in the regulation of solvent and spore formation. Signal molecule accumulation appears to be an important parameter that, together with other environmental and internal stimuli, is sensed and integrated by a complex regulatory network to govern fermentation metabolism, sporulation and potentially other important aspects of the organism’s life cycle.

## Methods

### Bacterial strains and media

The bacterial strains utilized in this study are listed in Table S2. *C. acetobutylicum* ATCC 824 and its mutant derivatives were grown at 37 °C in an anaerobic cabinet (MG1000 Anaerobic Work Station, Don Whitley Scientific) containing an atmosphere of 80 % nitrogen, 10 % hydrogen and 10 % carbon dioxide. The organism was routinely cultured in supplemented clostridial basal medium (CBMS) [43], unless stated otherwise. CBMS was based on CBM as previously described [44], but contained glucose (50 g l^−1^) and calcium carbonate (5 g l^−1^) as a buffering agent. For agar plates, 10 g l^−1^ agar was added and calcium carbonate was omitted. *Escherichia coli* TOP10 was grown in lysogeny broth at 37 °C. Antibiotics were used at the following concentrations: chloramphenicol, 25 µg ml^−1^; erythromycin, 40 µg ml^−1^; tetracycline, 10 µg ml^−1^; thiamphenicol, 15 µg ml^−1^. *C. acetobutylicum* wild-type and all mutants generated in this study were stored as spore stocks.

### Plasmids, oligonucleotides, DNA techniques

The plasmids and oligonucleotides used in this study are listed in Tables S3 and S4 and were synthesized by Eurofins MWG Operon, Germany. PCR amplifications were carried out using high-fidelity Phusion polymerase or *Taq* DNA polymerase (both from New England Biolabs). Electroporation of *C. acetobutylicum* was performed as described previously [14]. Plasmid isolation and genomic DNA preparations were carried out using the QIAprep Miniprep kit (Qiagen, UK) and DNeasy Blood and Tissue kit (Qiagen), respectively. Restriction enzymes were supplied by New England Biolabs and Promega and were used according to the manufacturers’ instructions. Southern blotting and hybridization was carried out as previously described [43].

### Construction of mutants using ClosTron technology

Mutants were constructed in *C. acetobutylicum* ATCC 824 using retargeted ClosTron plasmids according to Heap *et al*. [29]. The plasmids were designed using the ‘intron targeting and design tool’ available on http://www.ClosTron.com/ClosTron2.php and purchased from DNA2.0. Numbers in the plasmid names (Table S3) indicate the retargeting site used, which, in the case of RRNPP-type genes, was located within the HTH-encoding region. Genomic DNA from putative mutants was subjected to several PCR screens to establish whether the ClosTron-derived group II intron had inserted into the desired gene target. These screens included (i) primer pairs that annealed on either side of the target site and (ii) individual flanking primers together with a group II intron specific primer (the latter amplifying the intron–exon junctions). The generated PCR fragments were sequenced to obtain definite proof that insertion had occurred at the desired position. Finally, using chromosomal DNA of all mutants, Southern blot analysis was performed to confirm that only single ClosTron insertions had occurred. At least three independent mutants were generated for each gene. This was done to avoid accidental isolation of mutant clones carrying undesired second-site mutations: *C. acetobutylicum* is known to spontaneously ‘degenerate’, resulting in strains with a reduced or abolished capacity to form solvents and heat-resistant endospores. A preliminary analysis revealed that one of the independently obtained *qsrC* mutant clones differed phenotypically from the other three and showed signs of degeneration (data not shown). This clone was therefore excluded from further phenotypic screening.

### Generation of complementation and overexpression vectors

To construct the *qsrB* complementation vector pMTL85141-*qsrB*, a 1659 bp fragment containing the *qsrB* gene and a 351 bp 5′ non-coding region expected to contain the gene’s native promoter were PCR-amplified from genomic *C. acetobutylicum* ATCC 824 DNA using the primer pair QsrB_C_F1/QsrB_C_R1 (Table S4). These contained SbfI and NotI restriction sites, respectively, so that the resulting fragment could be cloned into the equally digested clostridial shuttle vector pMTL85141 [30]. The resulting vector pMTL85141-*qsrB* was confirmed by restriction analysis and sequencing.

To obtain an overexpression vector in which *qsrB* expression was driven by the strong *C. sporogenes fdx*-promoter, the 1336 bp *qsrB* gene was PCR-amplified from genomic DNA with the primer pair QsrB_C_F2/QrB_C_R2 (Table S4). These primers contained NdeI and BamHI restriction sites, respectively, which were used to clone the obtained DNA fragment into the clostridial shuttle vector pMTL85143 downstream of the *fdx*-promoter (Dr Ying Zhang, University of Nottingham, unpublished). The resulting vector pMTL85143-*qsrB* was confirmed by restriction analysis and sequencing.

To obtain the *qspB* expression vector pMTL85143-*qspB*, the 178 bp *qspB* gene was PCR-amplified from genomic DNA of *C. acetobutylicum* ATCC 824 using the primer pair QspB_C_F1/QspB_C_R1. These primers contained NdeI and EcoRI restriction sites, respectively, which were used to clone the obtained DNA fragment into the clostridial shuttle vector pMTL85143 downstream of the *fdx*-promoter. The resulting vector pMTL85143-*qspB* was confirmed by restriction analysis and sequencing.

### Spore assays and detection of granulose

*C. acetobutylicum* strains were grown in 5 ml CBMS to enable sporulation. After 7 days, a 200 µl sample of culture was heated to 80 °C for 10 min. Serial dilutions were carried out and 20 µl aliquots of the heat-treated cell suspension plated onto CBM agar. Agar plates were incubated for 48 h before c.f.u. were enumerated. For each assay, a *spo0A* mutant [25] and the wild-type were included as negative and positive controls, respectively. Spore assays in the presence of QspB peptide derivatives were carried out as described above, with the following modifications: CBMS was inoculated to OD 0.05 with a *C. acetobutylicum* pMTL85143-*qsrB* preculture and grown for 4 h. At this point, 10 ml aliquots of the culture were distributed into individual 15 ml Falcon tubes, each containing 5 µl of a particular 20 mM peptide stock solution.

To assess the accumulation of granulose, *C. acetobutylicum* strains were grown on CBM agar containing 5 % glucose. Colonies were stained with iodine as previously described [14].

### Determination of colony size

Overnight cultures were serial diluted before plating onto CGM agar plates (clostridial growth medium containing 1.5 % agar [45]) and further incubation for 24 h. To avoid negative impacts on growth, the CGM plates did not contain antibiotics. Measurements were taken from enlarged plate images alongside a scale. For each colony, three independent diameter readings were taken and averaged to account for the fact that some colonies were noncircular. A total of 20 colonies per strain were analysed.

### Addition of synthetic QspB fragments to *qsrB*-overexpressing strains

Synthetic linear peptides representing C-terminal fragments of the QspB sequence were synthesized and purified by Peptide Protein Research Ltd (Fareham, UK). Thirteen variants were obtained initially (Fig. S5), the purity of which was estimated to range from 89–99 %, apart from peptides TRSLLGAE, LGAEPTWGWNISKLLF and TRSLLGAEPTWGWNISKLLF (72, 79 and 83 %, respectively). A selection of peptides showing the highest activities in an initial screen as well as several additional variants (as listed in Fig. 6) were then resynthesized to >95 % purity. Lyophilized peptides were dissolved in DMSO to obtain 20 mM stock solutions. Two hundred millilitres of CBMS was inoculated to OD 0.05 with a *C. acetobutylicum* pMTL85143-*qsrB* preculture and grown for 4 h. At this point, 10 ml aliquots of the culture were distributed into individual 15 ml Falcon tubes, each containing 5 µl of a particular 20 mM peptide stock solution. Controls only contained 5 µl DMSO. Each peptide or control culture was set up in triplicate.

### Analysis of fermentation products

*C. acetobutylicum* ATCC 824 wild-type and mutants were grown in 50 ml Falcon tubes containing 30 ml of CBMS. At relevant time points, 1 ml samples were removed, placed on ice and centrifuged at 16 000 ***g*** for 5 min to obtain cell-free culture supernatant. Extraction of fermentation products and their gas chromatographic analysis was carried out as described previously [43].

### Bioinformatics and statistical analyses

Amino acid sequence identity and similarity for RRNPP-type regulators was determined following their alignment over the entire sequence using clustal Omega (https://www.ebi.ac.uk/Tools/msa/clustalo/; default settings apart from five combined iterations). Outputs in FASTA format were used to calculate identity and similarity using SIAS (http://imed.med.ucm.es/Tools/sias.html; BLOSUM62, costs of 10 and 0.5 for creating and extending a gap, respectively; compared to length of smallest sequence). Phylogenetic trees were generated using the advanced workflow option on NGPhylogeny.fr (https://ngphylogeny.fr; MAFFT for multiple alignment, BMGE for alignment curation via, tree inference via PhyML+SMS, tree rendering: Newick display). TPRpred (https://toolkit.tuebingen.mpg.de/tools/tprpred) was used to establish the presence of putative Tetratricopeptide repeat domains [28].

All numerical data were stored and analysed in IBM SPSS Statistics 19 and 20 (IBM Corporation, Armonk, USA) and Microsoft Excel 2007 and 2010. Significance levels were determined with an independent sample two-way *t*-test in IBM SPSS Statistics (IBM). Data were graphically visualized in GraphPad Prism 5 (GraphPad Software, La Jolla, USA) and Excel. The error bars provided indicate standard deviation.

## Supplementary Data

Supplementary material 1
