# Supplementary material for: RRNPP-type quorum sensing affects solvent formation and sporulation in Clostridium acetobutylicum
Source: Microbiology (Reading). 2020 Apr 28;166(6):579–92. doi: 10.1099/mic.0.000916 (PMC7376267; doi:10.1099/mic.0.000916)
Supplement: Supplementary material 1 [file mic-166-579-s001.pdf]

Figure S1

A)

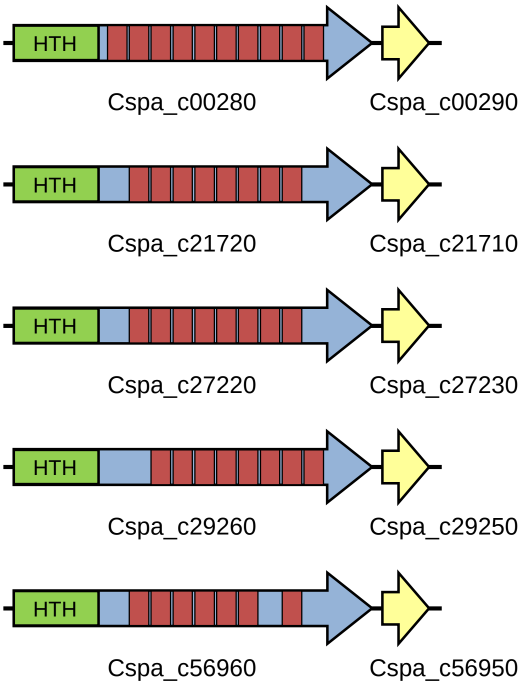

B)

positively charged — **hydrophobic** — **predicted mature signalling peptide**

```
Cspa_c29250     MKKKLIKGV--SLFLSC--FALTLVLNATTA----KTNNSNPAKVHLDESAPWG- 46
Cspa_c27230     MKKLLLTMVACVVLLSNVAFAATKTTSTTTDTKSTITS-SSGEKFHLDSRDPDGW 54
Cspa_c00290     MKKIILAVIATSMLLTNVAFASTKTTHKNT----NSTKVTQNNVYSVNSIDPH-- 49
Cspa_c21710     MKKIILAVMATSMLLTNVAFASTKTTHTKITKNSTVSSVDPNNVYHLNTLDPN-- 53
Cspa_c56950     MKKIILAVIATSMLLTNVAFASTKTTHTNTGKNTTVSSVKQNSMYHLNTTDPY-- 53
                *** ::  :   ::*:   ** * .   .       :.        ::   *
```

Figure S1. Schematic representation of *C. saccharoperbutylacetonicum* RNPP quorum sensing gene clusters (A) and alignment of putative signalling peptide precursor sequences (B).

(A) Four RNPP quorum sensing gene clusters have been identified, each encoding an RNPP-type regulator (large arrows) and a signalling peptide precursor (short yellow arrows). The locus tags for each system are provided. Regions encoding a putative helix-turn-helix motif (HTH, green) and tetratricopeptide repeat domains (red) are indicated. (B) The Clustal Omega amino acid sequence alignment shows the four predicted Qsp proteins. A conserved proline in the C-terminal region is indicated with red font; blue and green fonts indicate positively (K, R) and negatively charged (D, E) amino acids, respectively. Identical (*), conserved (:), and semi-conserved substitutions (.) are shown. Numbers indicate the length of the different precursor proteins. Positively charged, hydrophobic, and predicted signalling peptide-encoding regions are indicated with blue, grey, and red lines, respectively.

Figure S2

A)

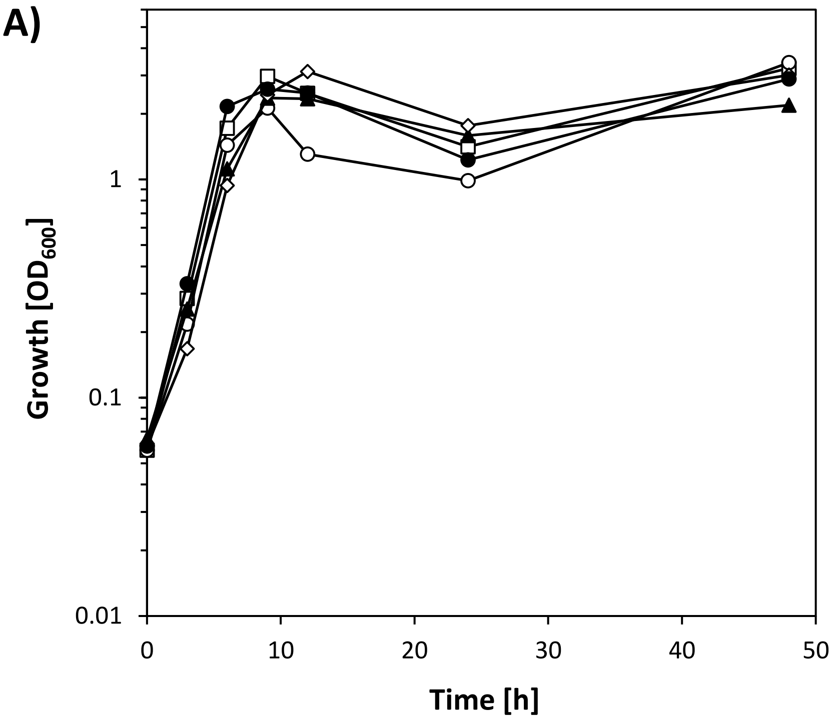

B)

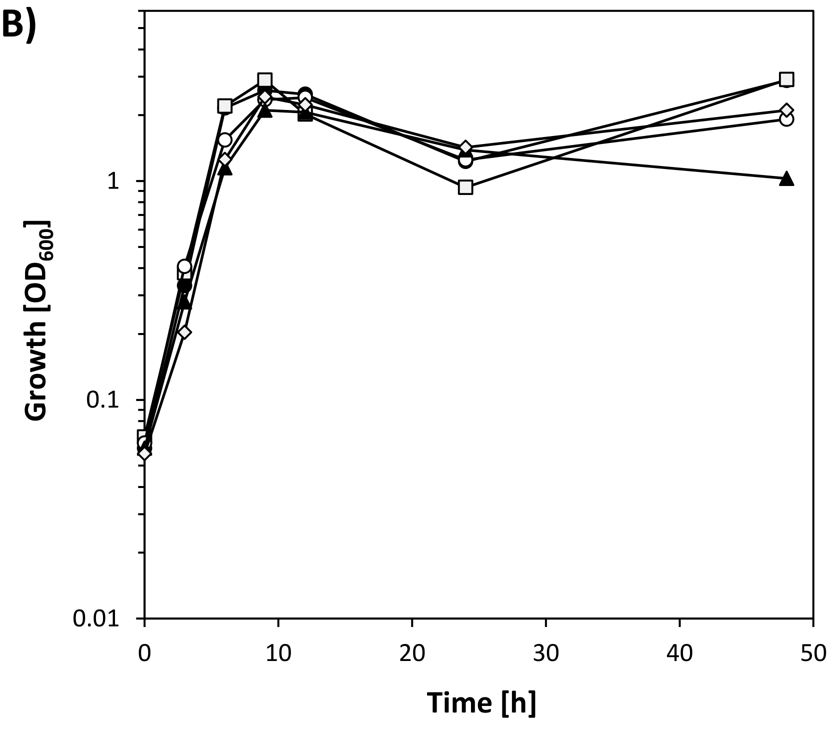

Figure S2. Growth of *C. acetobutylicum* ATCC 824 and derived *qsr* mutants in CBMS medium.

The data represent the mean of three independently cultures. A) Wild type (closed circles); *qsrA* (open squares), *qsrB* (open circles), *qsrC* (closed triangles) and *qsrD* (open diamonds) mutants. B) Wild type (closed circles); *qsrE* (open squares), *qsrF* (open circles), *qsrG* (closed triangles) and *qsrH* (open diamonds) mutants.

Figure S3

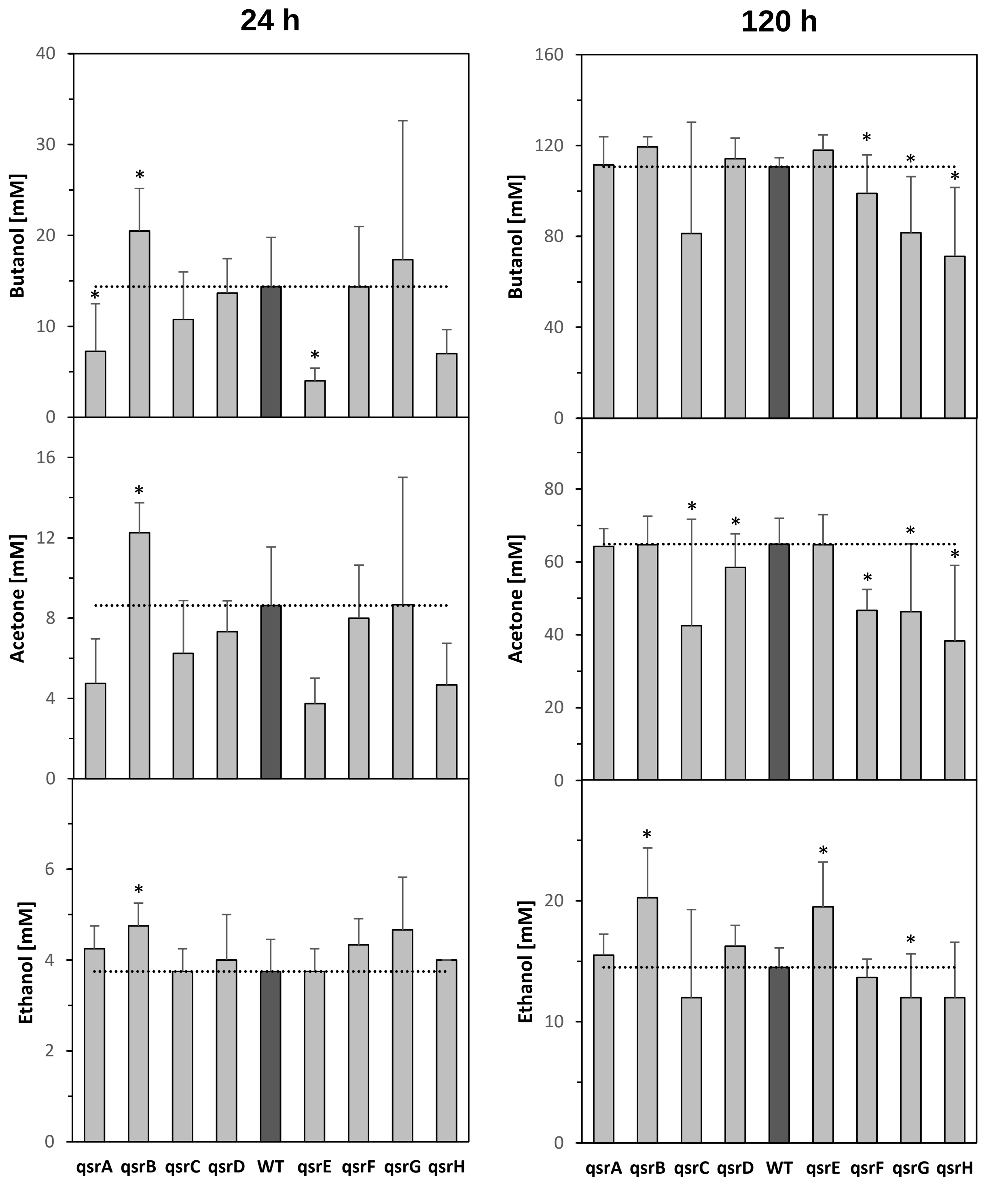

Figure S3. Solvent formation by *C. acetobutylicum qsr* mutants.

Formation of butanol (A), acetone (B) and ethanol (C) was monitored in CBMS broth after 24 h (left hand panels) and 124 h (right hand panels) for all eight *qsr* mutants and compared to the ATCC 824 parent strain. (B) After 72 h, culture supernatant samples were taken and analysed for the produced acids (acetate, checks; butyrate, lines) and solvents (butanol, white; acetone, grey; ethanol, black). The data represent the mean of three independent CBMS cultures with error bars indicating the standard deviation. Significant differences ($p \leq 0.05$) compared to the wild type are indicated by an asterisk.

Figure S4

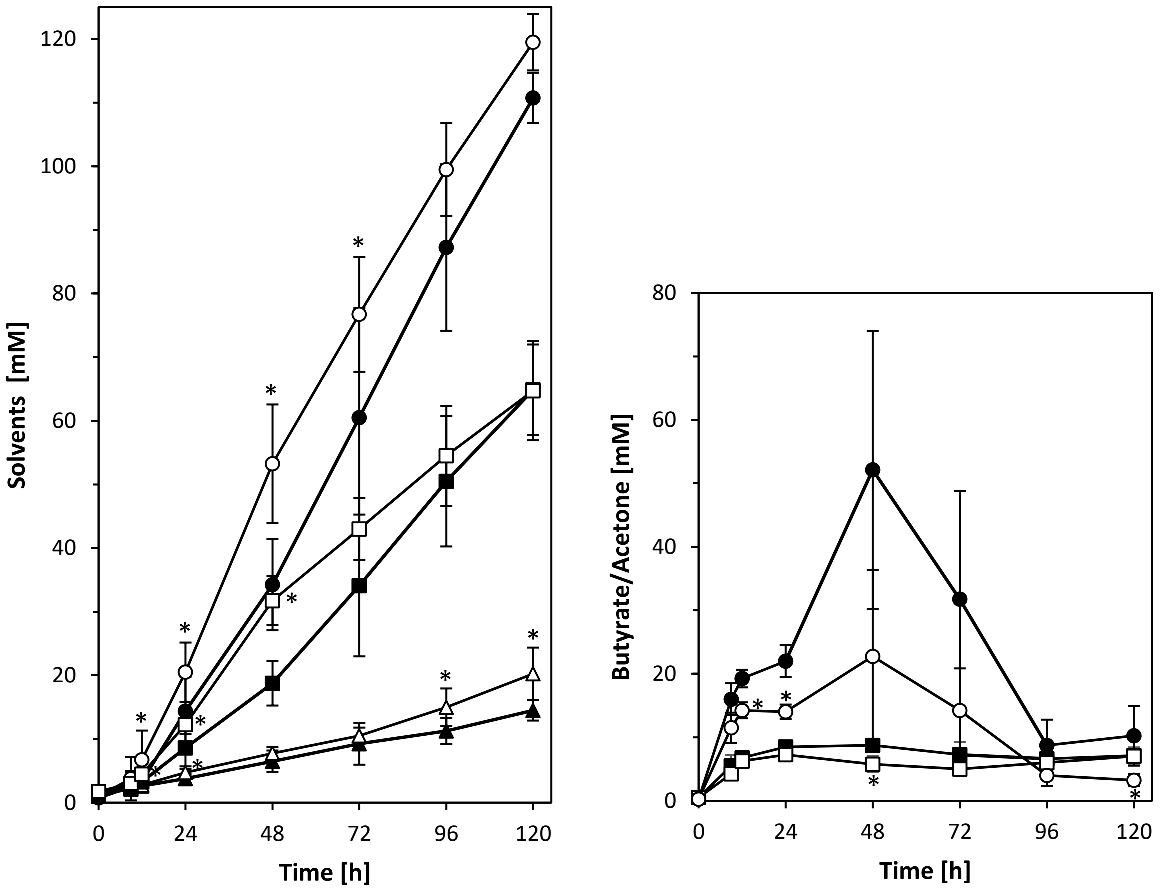

Figure S4. Solvent and acid production by *C. acetobutylicum qsrB* mutants
(A) Concentration of butanol (circles), acetone (squares) and ethanol (triangles) in the culture supernatant at the indicated time points. Open and closed symbols represent *qsrB* mutant and ATTC 824 parent strain data, respectively. (B) Concentration of butyrate (circles) and acetate (squares) in the culture supernatant at the indicated time point. Open and closed symbols represent *qsrB* mutant and ATTC 824 parent strain data, respectively. Data represent the mean of three independent cultures with error bars indicating the standard deviation. Significant differences ($p \leq 0.05$) compared to the wild type are indicated by an asterisk next to the relevant data point.

Figure S5

Peptide	Peptide activity
MLPKKSNKFILVLILCAIVFVSAFALNTSGTRSL**L**GAE**P**TWG**W**NISKLLF	
TRSLLGAEPTWGWNISKLLF	++
SLLGAEPTWGWNISKLLF	+++
LGAEPTWGWNISKLLF	++
AEPTWGWNISKLLF	++
PTWGWNISKLLF	-
AEPTWGW	+++
LGAEPTWGW	+++
SLLGAEPTWGW	+++
TRSLLGAEPTWGW	+++
SLLGAE	-
TRSLLGAE	-
SGTRSLLGAE	-
NISKLLF	-

Figure S5. Synthetic peptides alleviate *qsrB*-mediated repression of solvent formation.

The indicated synthetic peptides dissolved in DMSO were added to CBMS cultures of *C. acetobutylicum* pMTL85141-*qsrB* after 4 h of growth to a final concentration of 10 µM. Equivalent DMSO controls were performed for *C. acetobutylicum* pMTL85141 and *C. acetobutylicum* pMTL85141-*qsrB*, respectively. Triplicate cultures were grown for 5 days and analysed for final butanol titres. The ability to overcome *qsrB*-mediated repression of butanol formation was scored in comparison to the DMSO controls as follows: -, no significant difference to the *C. acetobutylicum* pMTL85141-*qsrB* DMSO control*;* ++, final butanol levels 40-66% of the *C. acetobutylicum* pMTL85141 DMSO control; +++, final butanol levels 67-100% of the *C. aceto-butylicum* pMTL85141 DMSO control. *C. acetobutylicum* pMTL85141-*qsrB* DMSO and *C. acetobutylicum* pMTL85141 DMSO controls produced 14±8 mM and 123±17 mM of butanol, respectively. The complete QspB sequence is given at the top with the three conserved amino acid positions (leucine, proline, tryptophan) in the C-terminal region indicated by bold red lettering.

TABLE S1. Formation of heat resistant endospores by *qsr* mutants

Strain	Heat-resistant CFU/ml	p value[1]
C. acetobutylicum qsrA::CT*ermB*	9.50×10^{7}	0.561
C. acetobutylicum qsrB::CT*ermB*	1.18×10^{8}	0.929
C. acetobutylicum qsrC::CT*ermB*	7.57×10^{7}	0.323
C. acetobutylicum qsrD::CT*ermB*	8.91×10^{7}	0.473
C. acetobutylicum qsrE::CT*ermB*	1.26×10^{8}	0.743
C. acetobutylicum qsrF::CT*ermB*	8.22×10^{7}	0.339
C. acetobutylicum qsrG::CT*ermB*	3.85×10^{7}	*0.036
C. acetobutylicum qsrH::CT*ermB*	1.21×10^{8}	0.857
C. acetobutylicum ATCC 824	1.15×10^{8}	-

[1]Significant differences to the wild type are indicated by an asterisk.

Table S2. Bacterial strains used in this study

Strain	Relevant properties	Source/reference
E. coli Top10	F- *mcrA* Δ(*mrr-hsdRMS-mcrBC*) Φ80*lacZ*ΔM15 Δ*lacX*74 *recA*1 *araD*139 Δ(*ara leu*)7697 *galU galK rpsL* (StrR) endA1 nupG	Invitrogen
E. coli Top 10 pAN2	*E. coli* Top 10 with methylation plasmid pAN2 containing the ϕ3TI methyltransferase	Heap et al. (2007)
C. acetobutylicum ATCC 824	*C. acetobutylicum* ATCC 824 wild type	Prof. Hubert Bahl, University of Rostock (COSMIC-strain)
C. acetobutylicum qsrA::CT*ermB*	*C. acetobutylicum* ATCC 824 *qsrA* ClosTron mutant	This work
C. acetobutylicum qsrB::CT*ermB*	*C. acetobutylicum* ATCC 824 *qsrB* ClosTron mutant	This work
C. acetobutylicum qsrC::CT*ermB*	*C. acetobutylicum* ATCC 824 *qsrC* ClosTron mutant	This work
C. acetobutylicum qsrD::CT*ermB*	*C. acetobutylicum* ATCC 824 *qsrD* ClosTron mutant	This work
C. acetobutylicum qsrE::CT*ermB*	*C. acetobutylicum* ATCC 824 *qsrE* ClosTron mutant	This work
C. acetobutylicum qsrF::CT*ermB*	*C. acetobutylicum* ATCC 824 *qsrF* ClosTron mutant	This work
C. acetobutylicum qsrG::CT*ermB*	*C. acetobutylicum* ATCC 824 *qsrG* ClosTron mutant	This work
C. acetobutylicum qsrH::CT*ermB*	*C. acetobutylicum* ATCC 824 *qsrH* ClosTron mutant	This work
C. acetobutylicum qspB::CT*ermB*	*C. acetobutylicum* ATCC 824 *qspB* ClosTron mutant	This work
C. acetobutylicum pMTL85141	ATTC 824 wild type with empty pMTL85141 vector	This work
C. acetobutylicum pMTL85143	ATTC 824 wild type with empty pMTL85143 vector	This work
C. acetobutylicum qsrB::CT*ermB* pMTL85141	*qsrB* mutant with empty ATTC 824 wild type with empty pMTL85141 vector	This work
C. acetobutylicum qsrB::CT*ermB* pMTL85141-*qsrB*	Complemented *qsrB* mutant carrying pMTL85141-*qsrB*	This work
C. acetobutylicum qsrB::CT*ermB* pMTL85143	*qsrB* mutant with empty pMTL85143 vector	This work
C. acetobutylicum qsrB::CT*ermB* pMTL85143-*qsrB*	Complemented *qsrB* mutant carrying pMTL85143-*qsrB*	This work
C. acetobutylicum pMTL85141-*qsrB*	*qsrB* overexpressing ATTC 824 wild type carrying pMTL85141-*qsrB*	This work
C. acetobutylicum pMTL85143-*qsrB*	*qsrB* overexpressing ATTC 824 wild type carrying pMTL85143-*qsrB*	This work
C. acetobutylicum qspB::CT*ermB* pMTL85143	*qspB* mutant with empty plasmid	This work
C. acetobutylicum qspB::CT*ermB* pMTL85143-qspB	Complemented *qspB* mutant	This work
C. acetobutylicum pMTL85143-*qspB*	*qspB* overexpressing ATTC 824 wild type carrying pMTL85143-*qssB*	This work

Table S3. Plasmids used in this study

Plasmid	Relevant properties	Source
pAN2	Plasmid containing ϕ3TI methyltransferase	Heap et al. (2007)
pCR2.1-TOPO	A plasmid that is supplied linearized with A-overhangs for convenient cloning of PCR fragments	Invitrogen
pMTL007C-E2::*qsrA*-102|103A	ClosTron plasmid retargeted to *qsrA*[1]	This study
pMTL007C-E2::*qsrB*-102|103S	ClosTron plasmid retargeted to *qsrB*[1]	This study
pMTL007C-E2::*qsrC*-102|103S	ClosTron plasmid retargeted to *qsrC*[1]	This study
pMTL007C-E2::*qsrD*-49|50A	ClosTron plasmid retargeted to *qsrD*[1]	This study
pMTL007C-E2::*qsrES*-58|59A	ClosTron plasmid retargeted to *qsrE*[1]	This study
pMTL007C-E2::*qsrF*-107|108A	ClosTron plasmid retargeted to *qsrF*[1]	This study
pMTL007C-E2::*qsrG*-93|94A	ClosTron plasmid retargeted to *qsrG*[1]	This study
pMTL007C-E2::*qsrH*-58|59A	ClosTron plasmid retargeted to *qsrH*[1]	This study
pMTL007C-E2::*qspB*-53/54A	ClosTron plasmid retargeted to *qspB*[1]	This study
pMTL85141	Clostridium modular plasmid containing *catP*	Heap et al. (2009)
pMTL85143	pMTL85141 with *C. sporogenes* ferredoxin promoter upstream of multiple cloning site	Dr Ying Zhang, Univ. of Nottingham
pMTL85141-*qsrB*	pMTL85141 containing *qsrB* coding region and 351 bp non-coding region upstream	This study
pMTL85143-*qsrB*	pMTL85143 containing *qsrB* coding region	This study
pMTL85143-*qspB*	pMTL85143 containing *qspB* coding region	This study

[1]Numbers following the gene name indicate the predicted insertion site of the encoded ClosTron derivative, with S and A denoting sense and anti-sense orientation, respectively.

Table S4. Oligonucleotides used in this study

Oligonucleotide	Sequence (5' to 3')
**ClosTron mutant screening**	
QsrA_F	AAGAGGAATTAGCGGGAGCTGAG
QsrA_R	CGACTTCTGTCAATTTGGTTGAGAAGC
QsrB_F	CGATATTGTTGGAGAAGAAGTTACTC
QsrB_R	AGATAATCCGCAGTTACATCC
QsrC_F	TCAAATACTGCCTATTGGCGTAAAGC
QsrC_R	AGCATTATTTCTGCTGCATGTCTAG
QsrD_F	GGAGAGTTTTGTCATATGTGTGTC
QsrD_R	AGCTTGTGATTCCTCATCCTC
QsrE_F	GATAAGGGAGAAAGTGCTATGGCAAG
QsrE_R	TCCTCTTGAAAAGGCATCTCTCTT
QsrF_F	AGATGATATTGTAGGTACAGAACTCAC
QsrF_R	GTCCTGTATGTATGAGGCGATC
QsrG_F	ACGGCCTAAGTCAAGAAGATCTGG
QsrG_R	ATTGCTTGCGATTTCTCATCTTCCATC
QsrH_F	GCACTTATGAGATAATGTCTATTGGAGACAAGC
QsrH_R	TGCTGCACTTCTAGTAAGGTTTGCT
EBS universal	CGAAATTAGAAACTTGCGTTCAGTAAAC
**Cloning**	
QsrB_C_F1	TATATACCTGCAGGCTACATTACTCAAAGCATATAAATACG
QsrB_ C_R1	TATATAGCGGCCGCTTACTTAAACTTTATTAAAAAATTTAATATTTTATCT ATGTC
QsrB_ C_F2	CTTGGTCATATGGGAAACTGTC
QsrB_ C_R2	AACATCGGATCCTATTTACTTACTTAAAC
QspB_ C_F1	TGTCTACATATGTTACCAAAAAAGAGTAATAAATTTATATTAG
QspB_ C_R1	TTTTTAGAATTCGGTTTTTGTTTAATGTTATAAAAC
**Southern Blot probe generation**	
EBS2	TGAACGCAAGTTTCTAATTTCGGTTCTCATCCGATAGAGGAAAGTGTCT
Intron SalI-R1	ATTACTGTGACTGGTTTGCACCACCCTCTTCG
